# Efficacy of two doses of dexmedetomidine on attenuating cardiovascular response and safety of respiratory tract to extubation

**DOI:** 10.34172/jcvtr.2023.31647

**Published:** 2023-06-29

**Authors:** Hamidreza Shetabi, Shima Karimian

**Affiliations:** ^1^Anesthesiology and Critical Care Research Center, Isfahan University of Medical Sciences, Isfahan, Iran; ^2^Student Research Committee, Isfahan University of Medical Sciences, Isfahan, Iran

**Keywords:** Dexmedetomidine, Extubation, Hemodynamics

## Abstract

**Introduction:**

Extubation can be associated with an adverse hemodynamic or respiratory response, which may be serious in cardiovascular written or in the elderly. The present study was conducted with the aim of investigating the effect of two different doses of dexmedetomidine in the prevention of extubation complications.

**Methods:**

This randomized clinical trial was conducted in Isfahan in 2020-2021 on 174 patients undergoing elective surgery. Patients were randomly divided into 3 groups receiving dexmedetomidine 1 μg/kg (D1), dexmedetomidine 0.5 μg/kg (D2), and normal saline (S). Hemodynamic variables include heart rate (HR), systolic blood pressure (SBP), diastolic blood pressure (DBP), mean arterial pressure (MAP), and peripheral blood oxygen saturation (Spo2) was measured and recorded before removing the endotracheal tube and at 1, 3, 5 and 10 minutes after extubation. Also, airway responses to extubation such as cough, hoarseness, and laryngospasm were investigated.

**Results:**

SBP, MAP, and HR in the D1 group were significantly lower than in other groups. In the D2 group, these measurements were lower than the control group at 3, 5, and 10 minutes after extubation (*P*<0.05 for all). In placebo group, SBP, MAP, and HR increased significantly after extubation (*P*=0.01). In group D1, cough (*P*=0.007) and its intensity (*P*=0.013), nausea and vomiting (*P*=0.04) and chills (*P*=0.001) were less than in other groups.

**Conclusion:**

In the D1 group, attenuation of autonomic response to extubation was more than other groups and side effects were less than D2 group, and in both groups, these side effects were less than the saline group.

## Introduction

Laryngoscopy and endotracheal intubation are two main procedures in patients undergoing surgical operations requiring general anesthesia.^1^ This procedure is also conducted in patients to avoid possible aspiration. Endotracheal intubation involves inserting a flexible plastic tube into the trachea that provides a suitable airway for mechanical ventilation. The most common method for intubation is via a laryngoscope.^2,3^

Extubation is conducted when the patient no longer requires mechanical ventilation and is usually conducted in the operation room or the recovery.^4^ This procedure is done by extracting the endotracheal tube and is associated with discomfort in patients.^5^ Coughing, respiratory depression, nausea, vomiting, and vocal cord injuries could occur after the intubation.^6^ Due to these discomforts, the patients might also experience changes in the hemodynamics that could be harmful in different conditions especially when the patient has undergone cardiopulmonary surgeries.^7^

As a result, developing a prophylactic and therapeutic strategy for these complications has high clinical value.^8^ Different medications have been used to prevent hemodynamic instability and complications after extubation in patients. Sedatives and analgesics have shown high efficacy in this issue.^9-11^

Dexmedetomidine is an anxiolytic, sedative, and pain medication. Dexmedetomidine is notable for its ability to provide sedation without risk of respiratory depression (unlike other commonly used drugs such as propofol and fentanyl) and can provide cooperative or semi- arousable sedation.^12^ Dexmedetomidine is commonly used in intensive care units (ICU) for sedation of intubate and under mechanical ventilation patients.^13^ The use of this drug is also reported to be associated with stability during different surgical operations.

Based on the evidence, dexmedetomidine could continuously be infused in mechanically ventilated patients before, during, and after extubation. Researchers believe that this drug might also be useful for the prevention of extubation complications including hemodynamic instability. ^14^

 Due to the importance of the extubation procedure and the prevalence of different complications and giving attention to the values of preventive strategies for these complications, in the present study, we aimed to investigate and compare the effects of dexmedetomidine in two different dosages in the prevention of extubation complications in patients.

## Materials and Methods

 This is a double-blind randomized clinical trial that was conducted in 2019-2019 at Al-Zahra Hospital. After receiving the Ethics Code IR.MUI.MED.REC.1399.954 from the Isfahan University of Medical Sciences and registered in the Iranian clinical trial center with ID IRCT20180416039326N19, the present study was conducted on 180 patients who were candidates for elective open cholecystectomy in supine position under general anesthesia. The inclusion criteria were age between 18 to 60 years, American Society of Anesthesiologists (ASA) classification 1 or 2, Body mass index of ≤ 30 kg/m2, and signing the written informed consent to participate in this study. Patients with the following conditions did not enter the study: history of allergy to alpha-adrenergic drugs, preoperative bradycardia (HR < 50/min) or arrhythmias, grade 2 or 3 of the atrioventricular block (AV block), vascular problems, hypovolemia, the use of vasodilator, uncontrolled diseases (liver or kidney diseases, diabetes mellitus, and seizure), addiction to drugs and substances, smoking, airway infection or history of airway surgery, patients at risk for increased intracranial pressure and intraocular pressure, the use of digoxin and gabapentin, incomplete data and transferring the patients to the ICU before extubation.

 Sampling was easy and accessible, sample size was calculated using the formula of estimating the sample size to compare the means and assuming a = 01 0.01 and b = 0.1, according to the following formula and considering the success of 64% and 29% in groups receiving doses of 0.5 and 1 µg/kg of body weight. The required number of samples was obtained for 60 people in each group or 180 patients in all three groups.


$$n^{\prime }=\left[\frac{\left(z_{1-a/2}\sqrt{2\bar{p}\left(1-\bar{p}\right)}+z_{1-\beta }\sqrt{p_{1}\left(1-p_{1}\right)}+p_{2}\left(1-p_{2}\right)\right)}{p_{1}-p_{2}}\right]$$

Patients were randomly assigned to three equal groups using Random Allocation software. Demographic information of all cases including age, sex, weight, BMI, ASA classification, and presence of any comorbidities were collected. All patients underwent electrocardiogram monitoring, non-invasive intermittent sphygmomanometer, pulse oximetry, and capnography upon entering the operating room. Premedication with midazolam 2mg and ondansetron 4 mg IV then preoxygenation with 100% oxygen for 3 min was done. Induction of anesthesia is performed with propofol (2 mg/kg), fentanyl 2 µg/kg, and then atracurium (0.5 mg/kg) then intubation was conducted using an appropriate endotracheal tube. Maintenance of anesthesia was with 50% oxygen and nitrous oxide, Isoflurane 1-1.2 MAC, and 0.1 mg/kg morphine. With the start of the surgical suture, the studied drugs were injected in the same volume by an anesthesiologist who was not a member of the research team.

 The first group (D1) received 1 µg/kg dexmedetomidine in 100 ml normal saline intravenous

 The second group (D2) received 0.5 µg/kg dexmedetomidine in 100 ml normal saline intravenous

 The third group(S) received the same volumes of normal saline as a placebo. The infusion of the contents of the syringes (dexmedetomidine and normal saline) was done within 10 minutes.

 At the end of the surgery, the anesthetic gases were cut off and the patients received 3 liters of oxygen per minute and the extubation was performed. When patients opened their eyes, they were encouraged to take deep breaths, and after spontaneous breathing, the endotracheal tube was removed by confirming the current volume and proper ventilation frequency. Immediately after removing the endotracheal tube, patients received 5 liters of oxygen per minute through a face mask.

 Hemodynamic variables including heart rate (HR), systolic blood pressure (SBP), diastolic blood pressure (DBP), mean arterial pressure (MAP), and peripheral blood oxygen saturation before removal of the tracheal tube and 1, 3, 5, and 10 minutes after extubation were measured and recorded. Also, the length of stay in recovery and the time of tube removal were evaluated for each patient. In this study, the data collector was blind to the groups

Furthermore, the frequency of coughing after extubation and its severities were measured. The severity of coughing was based on a modified 4-point Minogue scale: 15 grade 1, no cough; grade 2 (mild), coughing once or twice; grade 3 (moderate), fewer than 4 non-sustained coughs lasting 1–2 s each or overall coughing lasting less than 5 s; grade 4 (severe), at least 4 coughs lasting at least 2 s, or overall coughing. We also collected other complications following extubation including laryngospasm after extubation, respiratory depression, nausea and vomiting, delirium, shivering, and hoarseness. The obtained data were entered into the Statistical Package for Social Sciences (SPSS) version 24. Quantitative data were reported as mean ± standard deviation and qualitative data as frequency distribution (percentage). Independent t-test, Chi-square, and repeated measure test were used to analyze the data. *P* value < 0.05 was considered as a significance threshold.

## Results

**Figure 1:**
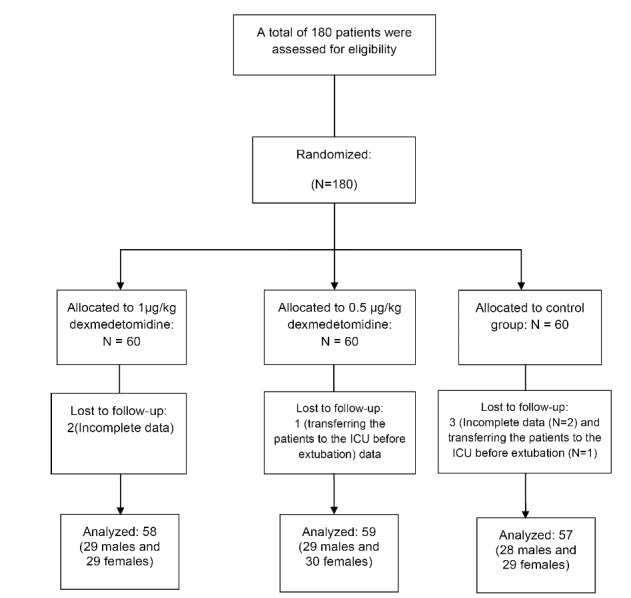
CONSORT flow diagram of study

In the present study, a total number of 180 patients entered the study and were randomized into 3 groups each containing 60 patients. During the study, 2 patients in the D1 group, 1 patient in the D2, and 3 patients in the S group were excluded due to incomplete data and transferring the patients to the ICU before extubation. Data from 174 patients were analyzed. Flowchart of the patients selection (CONSORT flow diagram) is illustrated in Figure 1.

Analysis of demographic data between the three groups showed no significant differences between 3 groups regarding age (*P* = 0.20), gender (*P* = 0.41), weight (*P* = 0.51), BMI (*P* = 0.13), ASA classification (*P* = 0.32), and the presence of controlled and under-treatment comorbidities (including liver or kidney diseases, diabetes, and seizures) (*P* = 0.22).

Data are summarized in Table 1.

**Table 1:** Analysis of demographic data between three groups.

**Variable**	**Group D1**	**Group D2**	**Group S**	***P *** **value**
Age (yeas) (mean ± SD)	43.32 ± 11.50	45.68 ± 10.29	44.92 ± 11.32	0.20
Weight (kg) (mean ± SD)	72.29 ± 9.23	72.72 ± 9.57	72.51 ± 8.83	0.51
Height (m) (mean ± SD)	171.24 ± 11.61	172.72 ± 12.42	172.07 ± 11.58	0.31
BMI (kg/m^2^) (mean ± SD)	23.94 ± 1.28	24.60 ± 1.60	24.33 ± 1.82	0.13
Gender (N %)Female	29 (50%)	30 (50.8%)	29 (50.8%)	0.41
Male	29 (50%)	29 (49.2%)	28 (49.2%)	
ASA (N %))1	42 (72.4%)	44 (74.5%)	42 (73.6%)	0.32
2	16 (27.6%)	15 (25.5%)	15 (26.4%)	
Comorbidities (N %))	15 (25.8%)	15 (25.4%)	13 (22.8%)	0.22
	43 (74.2%)	44 (74.6%)	44 (77.2%)	

 ASA: American Society of Anesthesiologists Group D1: Received 1 µg/kg dexmedetomidine

 Group D2: Received 0.5 µg/kg dexmedetomidine Group S: Received normal saline

Based on the results, the systolic blood pressure in group 1 D was significantly lower compared to other groups, and in group D 2, systolic blood pressure was lower compared to the control group at 3, 5, and 10 minutes after injection (*P* < 0.05 for All). Patients who received placebo had a significant increase in SBP after extubation (*P* = 0.01).

Based on our analyses, the patients that received 1µg/kg dexmedetomidine had a 13.5% reduction in their SBP, the patients that received 0.5µg/kg dexmedetomidine had a 10.9% reduction in their SBP and the control group had 6.8% reduction. These data are suggestive of the better efficacy of 1µg/kg dexmedetomidine. Evaluation of DBP showed no significant changes in DBP of the control group (*P* = 0.15) and these patients had significantly higher DBP within 3, 5, and 10 minutes after extubation compared to D1 and D2 groups (*P* < 0.05), but no differences were observed between the two D1 and D2 groups regarding DBP (*P* > 0.05).

During the study, we observed a significant decrease in MAP of both the D1 and D2 groups (*P* = 0.01 for both) and a significant increase in MAP of the control group (*P* = 0.03). MAP of both intervention groups was significantly lower at 5 and 10 minutes after drug administration (*P* < 0.05) (Table 2).

**Table 2:** Comparison of systolic, diastolic blood pressure and MAP between groups.

**Group**		**T1**	**T2**	**T3**	**T4**	**T5**	**P**_time_	**P**_time*intervention_	**P**_intervention_
D1(SBP)	Mean	138.05	120.25	113.10	100.54	98.80	0.001	0.32	0.64
	S.D	19.50	16.59	12.64	18.41	14.51			
D2 (SBP )	Mean	141.24	121.27	117.06	105.59	104.98	0.001		
	S.D	16.96	10.01	14.12	12.54	9.51			
S (SBP)	Mean	139.21	138.20	142.18	144.67	145.31	0.01		
	S.D	17.11	15.10	16.32	16.12	15.67			
P1		0.43	0.07	0.03	0.01	0.01			
D1(DBP)	Mean	93.40	81.24	72.10	67.28	64.13	0.001	0.08	0.14
	S.D	16.21	14.14	11.38	11.27	13.83			
D2 (DBP)	Mean	94.87	80.94	74.26	70.47	67.57	0.001		
	S.D	13.68	10.94	12.92	9.64	9.06			
S (DBP)	Mean	93.25	95.14	94.32	93.55	92.18	0.15		
	S.D	14.25	13.22	11.09	10.21	9.65			
P1		0.27	0.05	0.03	0.01	0.01			
D1(MAP)	Mean	128.01	112.54	104.94	99.36	98.07	0.001	0.37	0.28
	S.D	14.93	9.40	11.81	9.70	9.53			
D2 (MAP)	Mean	124.87	107.98	103.87	97.34	95.45	0.001		
	S.D	16.95	17.97	12.39	13.215	11.04			
S (MAP)	Mean	124.69	125.18	128.27	130.40	131.36	0.03		
	S.D	14.26	16.12	14.08	14.77	13.74			
P1		0.66	0.07	0.05	0.04	0.01			

T1: Before removing the endotracheal tube, T2: within 1 minute, T3: within 3 minutes, T4: within 5 minutes, T5: within 10 minutes. P_1: _Resulted from One-Way ANOVA for comparing mean pain score between three groups at each follow up time point. P_intervention_ indicates overall difference between three groups over the follow up period. P_time: _indicates the change in mean pain score in each intervention group over the follow up period. P_time*intervention_: indicates the interaction between time and intervention. P_intervention_, P_time, _and P_time*intervention _obtained from repeated measures ANOVA

 We also compared the pulse rate and SPO2 between the groups, the pulse rate in group D1 was lower compared to other groups, and the patients in group D2 had a lower heart rate than the control group within 5, 10, and 15 minutes after the extubation(P < 0.05 for all).

The HR was increased significantly in the placebo group after the extubation. No significant differences were observed between groups regarding SPO2 and hart rate (Table 3).

**Table 3:** Comparison of pulse rate and SPO2 between groups.

**groups**		**T1**	**T2**	**T3**	**T4**	**T5**	**P**_time_	**P**_time*intervention_	**P**_intervention_
D1 (HR)	Mean	85.01	80.07	71.56	62.909	61.68	0.001	0.157	0.241
	SD	15.47	13.91	17.05	11.91	12.93			
D2 (HR)	Mean	84.10	79.96	75.49	68.47	65.38	0.001		
	SD	13.11	14.58	12.65	12.75	13.27			
S (HR)	Mean	83.78	84.51	86.28	88.20	89.34	0.001		
	SD	12.69	12.84	11.44	12.84	12.84			
P1		0.43	0.15	0.68	0.02	0.01	0.03		
D1 (SPO2)	Mean	97.60	98.54	98.81	99.01	98.76	0.001	0.074	0.26
	SD	2.90	1.54	1.09	1.17	1.10			
D2 (SPO2)	Mean	97.89	98.70	98.85	99.21	98.89	0.001		
	SD	2.38	1.34	1.00	1.06	0.97			
S (SPO2)	Mean	98.88	98.75	98.82	99.02	99.04	0.001		
	SD	2.33	2.44	1.20	1.62	0.99			
P1		0.27	0.34	0.32	0.27	0.20	0.18		
D1 (SPO2)	Mean	85.01	80.07	71.56	62.909	61.68	0.001	0.157	0.241
	SD	15.47	13.91	17.05	11.91	12.93			
D2 (SPO2)	Mean	84.10	79.96	75.49	68.47	65.38	0.001		
	SD	13.11	14.58	12.65	12.75	13.27			
S (SPO2)	Mean	83.78	84.51	86.28	88.20	89.34	0.001		
	S.D	12.69	12.84	11.44	12.84	12.84			
P1		0.66	0.15	0.68	0.02	0.01	0.03		

T1: Before removing the endotracheal tube, T2: within 1 minute, T3: within 3 minutes, T4: within 5 minutes, T5: within 10 minutes. P_1: _Resulted from One-Way ANOVA for comparing mean pain score between three groups at each follow up time point. P_intervention_ indicates overall difference between three groups over the follow up period. P_time: _indicates the change in mean pain score in each intervention group over the follow up period. P_time*intervention_: indicates the interaction between time and intervention. P_intervention_, P_time, _and P_time*intervention _obtained from repeated measures ANOVA

According to our data, the incidence of cough (P = 0.007) and its severity (p = 0.013), nausea and vomiting (p = 0.04) and chills (p = 0.001) in group D1 is lower than in group D2 and in both were lower than the saline group. Regarding other findings, no significant difference was found between the groups. Side effects in D1 group patients were less than D2 group and in both groups were less than saline group. These data are summarized in Table 4.

**Table 4:** Comparison of different variables between three groups.

**Variable**	**Group D1**	**Group D2**	**Group S**	***P *** **value**
Duration of surgery (minutes)(mean ± SD)	113.3 ± 46.6	100.5 ± 42.7	103.4 ± 39.4	0.99
Recovery time (minute) (mean ± SD)	43.32 ± 11.50	45.68 ± 10.29	44.92 ± 11.32	0.22
Extubation time(minute) (mean ± SD)	77.63 ± 11.38	76.28 ± 10.42	77.92 ± 10.70	0.31
Coughing after extubation (N (%))	171.24 ± 11.61	172.72 ± 12.42	172.07 ± 11.58	0.007
Coughing severity (mean ± SD)	2.82 ± 1.6	3.22 ± 1.0	3.58 ± 1.3	0.013
Laryngospasm after extubation (N (%))	0	0	0	1
Respiratory depression (N (%))	1 (1.7%)	1 (1.7%)	2 (3.5%)	0.27
Nausea and vomiting (N (%))	12 (20.6%)	20 (33.9%)	29 (50.8%)	0.04
Delirium (N (%))	4 (6.8%)	5 (8.4%)	7 (12.2%)	0.06
Shivering (N (%))	14 (24.1%)	16 (27.1%)	31 (54.3%)	0.001
Hoarseness (N (%))	8 (13.8%)	7 (11.9%)	8 (14.0%)	0.33

*P* < 0.05 statistically significant.

## Discussion

 Normal hemodynamic response after the extubation includes increased blood pressure and heart rate, associated with coughing, and possibilities of other complications. The use of different sedative agents could prevent these complications and decrease hemodynamic changes. In the present study, we investigated the effects of two dosages of dexmedetomidine for patients undergoing extubation. Based on the results of our study, patients that received 1µg/kg dexmedetomidine had significantly lower blood pressures, heart rate, and complications including coughing and the severity of coughs.

 On the other hand, administration of 0.5 µg/kg dexmedetomidine was associated with similar results but the effects of 1 µg/kg dosage were more effective. We also found that the patients that received a placebo had increased blood pressure in the study.

One of the findings of our study was that using 1µg/kg dexmedetomidine could prevent increasing hemodynamic changes such as blood pressure and heart rate after extubation. Different studies have been conducted in this regard that assessed the use of dexmedetomidine. In 2015, a study was performed by Gupta and colleagues in India on 40 adults. This study showed that administration of 0.2- 0.7 µg/kg dexmedetomidine resulted in significantly decreased heart rate and blood pressure after the extubation compared to midazolam. It was also shown that extubation duration in the dexmedetomidine group was significantly lower than in the midazolam^15^ In another study, Fan and colleagues indicated that usage of 0.7 µg/kg dexmedetomidine had better results compared to 0.5 µg/kg dexmedetomidine in lowering the blood pressures and prevention of hemodynamic changes in adults undergoing extubation but also mentioned that remifentanil had more significant results compared to both groups.^16^ Similar results were reported by another study, which showed that an infusion of 0.6 μg/kg/h of dexmedetomidine could significantly reduce blood pressure in patients, but had no significant effect on respiratory depression. ^17^ These data were in line with the findings of our study showing the effectiveness of dexmedetomidine for extubation. An important point that should be mentioned is that none of these studies could provide evidence on the best dosage for dexmedetomidine administration while here we compared dosages of 1 and 0.5 µg/kg and reported that injection of 1 µg/kg dosage was more effective than 0.5 µg/kg. We also found that the frequencies of coughing and the severity of coughs were significantly lower in patients receiving 1 µg/kg dexmedetomidine compared to other patients. Hu and others explained that both lidocaine and dexmedetomidine had equal effectiveness in attenuating cough and hemodynamic changes during the tracheal extubation period after thyroid surgery.^17^ In another study, it was indicated that the addition of a single dose (0.5 µg/kg) of dexmedetomidine during emergence from sevoflurane remifentanil anesthesia was effective in attenuating coughing and hemodynamic changes and did not exacerbate respiratory depression after thyroid surgery.^18^ It has been declared that prevention of coughing and reducing the severity of coughs after extubation could have high clinical importance because coughing could lead to serious complications after some surgical operations and as a result, much effort has been given to the development of preventive strategies.^19,20^ Recent studies have investigated medical prevention of coughs after extubation in cases with COVID-19 that undergo surgical operations and have indicated that these strategies could lead to significant prevention of medical staff infections.^4,21^ Based on our results, administration of 1 µg/kg dexmedetomidine resulted in significantly reduced coughing frequency and severity and this dosage was more effective.

 Among the limitations of this research was the small sample size and the exclusion of patients with a history of hypertension, smoking or drug addiction. Therefore, it is recommended to include these items in the future studies

## Conclusion

 The attenuation of the autonomic response to extubation in the dexmedetomidine 1 µg/kg group was more than that of the dexmedetomidine 0.5 µg/kg group. In patients receiving dexmedetomidine1µg/kg, the side effects were less than in the dexmedetomidine 0.5 µg/kg group and in both group lesser than in the saline group.

## Acknowledgements

 The authors appreciate the cooperation of the anesthesia and nursing staff of Al-Zahra Hospital, affiliated to Isfahan University of Medical Sciences.

## Competing Interests

 The authors declare no conflicts of interest.

## Ethical Approval

 The protocol of this study was approved by the ethics committee of the Isfahan University of Medical Sciences with the ethics code IR.MUI.MED.REC.1399.954.

## Funding

 This study was supported by a fund from the Vice Chancellor for Research of Isfahan University of Medical Sciences.
